# Investigating the feasibility and acceptability of a cognitive behavioural suicide prevention therapy for people in acute psychiatric wards (the ‘INSITE’ trial): study protocol for a randomised controlled trial

**DOI:** 10.1186/s13063-016-1192-9

**Published:** 2016-02-11

**Authors:** Gillian Haddock, Linda Davies, Emma Evans, Richard Emsley, Patricia Gooding, Lisa Heaney, Sarah Jones, James Kelly, Ailsa Munro, Sarah Peters, Daniel Pratt, Nicholas Tarrier, Kirsten Windfuhr, Yvonne Awenat

**Affiliations:** School of Psychological Sciences, Manchester University, Zochonis Building, Brunswick Street, Manchester, M13 9PL UK; Centre for Health Economics, Institute of Population Health, Manchester University, Jean McFarlane Building, Oxford Road, Manchester, M13 9PL UK; Manchester Mental Health and Social Care NHS Trust, Manchester, UK; Centre for Biostatistics, Institute of Population Health, Manchester University, 1.304 Jean McFarlane Building, Oxford Road, Manchester, M13 9PL UK; School of Psychological Sciences, Manchester University, Coupland Building 1, Oxford Road, Manchester, M13 9PL UK; Lancashire Care NHS Foundation Trust, Early Intervention Service, Accrington, UK; National Confidential Inquiry into Suicide and Homicide by People with Mental Illness, Centre for Mental Health and Safety, Manchester University, 2nd Floor, Jean McFarlane Building, Oxford Road, Manchester, M13 9PL UK

**Keywords:** Suicide, Cognitive therapy, Randomised controlled trial (RCT), Inpatients, Acute psychiatric wards, Self-harm, Qualitative

## Abstract

**Background:**

Suicide is a major cause of preventable death, and suicidal behaviour is prevalent in acute psychiatric wards. People admitted to acute psychiatric wards often experience repeated episodes of suicidal behaviour, causing great distress and heavy use of NHS services. There is little research investigating effective psychological treatments for suicidal patients in inpatient settings although previous research has found support for psychological therapies which specifically target suicidal behaviour. This paper describes the protocol of a single blind RCT to investigate the acceptability and feasibility of a cognitive behavioural intervention targeting suicidality (CBSP) for suicidal people in acute psychiatric wards.

**Methods/Design:**

A single blind RCT comparing treatment as usual (TAU) to TAU plus Cognitive Behavioural Suicide Prevention (CBSP) therapy (TAU + CBSP). Sixty participants (aged 18–65 years) who are suicidal, or have been within the past 3 months, will be recruited from NHS trusts in the North West of England. Our primary objective is to determine whether CBSP is feasible, acceptable and efficacious when compared to patients who receive TAU alone. Secondary aims are the impact of CBSP on suicidal thinking, behaviours, functioning, quality of life, service use and psychological factors associated with suicide. Assessments take place at baseline, 6 weeks and 6 months (end of treatment). The analysis will report on the feasibility and acceptability of CBSP. Qualitative data from staff and service users will inform feasibility and acceptability data.

**Discussion:**

Psychiatric inpatients are a high-risk group and the use of psychological therapies in these settings is rare and requires evaluation. This study is essential to investigate the unique contextual challenges involved in delivering psychological therapy to suicidal inpatients and to identify any necessary modifications required within inpatient settings. The findings will inform a larger, definitive trial.

**Trial registration:**

15 March 2012, PB-PG-1111-26026, NIHR ISRCTN17890126.

**Electronic supplementary material:**

The online version of this article (doi:10.1186/s13063-016-1192-9) contains supplementary material, which is available to authorized users.

## Background

Suicide is a major cause of preventable death. Between 2003 and 2013 in England, there were 49,251 deaths in the general population that received a suicide or ‘undetermined verdict’, i.e. a death from an undetermined cause at inquest, an average of 4477 per year [1]. In 2013, 6233 people died by suicide in the UK with 11.9 deaths from suicide per 100,000 people within the general population [2]. Seventy-eight per cent of all suicides in 2013 were male and suicide remains the leading cause of death for males aged between 20–34 and 35–49 [2].

It is estimated that 90 % of suicides are carried out by people with mental health difficulties [3] and many of these people are under the care of mental health services and have received inpatient care. In England, between 2002 and 2011, 63 % of patients who died by suicide had a mental health diagnosis [4]. During 2003 and 2013, there were 13,972 deaths (28 % of the general population suicides) identified as patient suicides (i.e. the person had been in contact with mental health services in the 12 months prior to death), which is an average of 1270 patient suicides per year [1]. In the same period, there were 1295 inpatient deaths by suicide (9 % of patient suicides), an average of 118 suicides per year. The first week of admission to a psychiatric inpatient ward is a high-risk period, with approximately one quarter of inpatient suicides occurring within this first week [5]. However, the post-discharge period has also been identified as a high-risk time for suicide among mental health patients, particularly the first few days after leaving hospital [6]. The National Confidential Inquiry into Suicide and Homicides reported 2368 suicides within 3 months of discharge from inpatient care (17 % of all patient suicides), which is an average of 215 suicides per year [1].

Suicidality also has a substantial economic impact. Treatment costs associated with suicidal behaviour are significant, with inpatient psychiatric care accounting for nearly 70 % of NHS costs [7]. In 2013–2014, over 105,000 patients received treatment in NHS psychiatric wards and readmission is commonplace [8]. Whilst the median length of stay was 23 days, at the end of 2013 more than 50 % of people in hospital have been there for over 117 days [8]. Current methods of recording clinical data do not identify the specific cost of treating suicidal behaviour in inpatient settings, so the true economic burden remains unclear [7]. There are economic benefits from delaying completed suicide as the number of lost years of productive activity will be reduced; overall it is estimated that averted costs are of £66,797 per year per person of working age where suicide is delayed [7].

In addition to completed suicides, suicidal behaviour is frequently a recurring experience rather than an isolated singular event [9, 10] and suicidality (referring to suicidal thoughts and behaviours) is often a reason for admission to an acute psychiatric ward. Given that suicidal behaviour is highly prevalent in acute psychiatric wards, and there is a high-risk period following discharge from inpatient wards, there is a need to identify treatments which reduce the likelihood of suicidality in these settings and in the post-discharge period.

Inpatient wards are known to be complex and challenging environments for staff and patients alike. Recent reports from the Royal College of Psychiatrists [11], the Care Quality Commission [12] and the Schizophrenia Commission [13] highlight low qualified staff/patient ratios, reliance on agency staff, lack of skills in care, and insufficient clinical supervision and training. This creates a custodial culture with limited opportunities for staff/patient engagement in therapeutic relationships focused on recovery. A recent study identified that 49 % of acute ward staff were emotionally exhausted, with 29 % showing psychological distress [14]. This highlights the significant challenges in implementing effective interventions in inpatient settings.

There are a number of local and national policies aimed at reducing suicide, particularly for people deemed at high risk, for example for people with mental health difficulties and for patients on acute psychiatric wards [15]. However, suicide rates and suicidal behaviours remain high and whilst there is a considerable body of research investigating the epidemiological risk factors for suicide, there is very little examining psychological treatment for suicidality in inpatient settings.

However, there is good evidence supporting the use of psychological treatments such as cognitive behaviour therapy (CBT) for suicidality in other settings [16, 17]. CBT has an extensive evidence base, has been shown to be efficacious and acceptable to patients and is a recommended intervention by NICE for a number of mental health conditions. In 2011, NICE released guidelines recommending CBT for longer-term treatment of self-harm [15]. Several approaches have been evaluated [17, 18]: however, none have focused on delivery within inpatient settings. Tarrier and colleagues [19] developed Cognitive Behavioural Prevention for Suicidality in Psychosis (CBSP), utilising the principles of CBT and targeting specific psychological processes that drive suicidality for use with complex clients. This therapy offers a structured intervention to address and amend the specific psychological architecture of suicidal behaviour. A community-based trial with people with schizophrenia showed that the treatment was successful in reducing suicidal ideation and suicide probability in a population with psychosis [17] and early findings from a further study involving suicidal prisoners look promising [20] and show CBSP to be feasible and acceptable with complex clients experiencing suicidal ideation.

CBSP is underpinned by the Schematic Appraisal Model of Suicide (SAMS) which is a dynamic model to help understand and guide psychological suicide prevention interventions [21]. The SAMS model explains how dysfunctional psychological processes lead to the formation of a ‘suicide schema’ (a core belief that becomes a habitual memory). Each episode of suicide crisis exerts a ‘kindling’ effect that strengthens the latent ‘suicide schema’, reinforcing more entrenched and elaborate plans. Each repetition increases the ease of schema activation, and, in turn, suicidal behaviour. This approach has not yet been evaluated with psychiatric inpatients experiencing suicidal behaviour.

Given that there are such high levels of suicidality within acute psychiatric wards and post discharge, there is a need to ascertain whether psychological treatments can be effective in reducing suicidality in this population. As there is growing evidence supporting the use of CBSP, the primary objective of the current trial is to explore whether CBSP is feasible and acceptable for service users and staff on acute inpatient psychiatric wards. Secondary objectives are to assess the impact of the intervention on suicide behaviour and ideation, functioning, quality of life, NHS service use and other psychological variables thought to be predictive of suicide. Qualitative data from staff and patients after the intervention will be used to assess feasibility and acceptability of the intervention and will help in the refinement of the approach in preparation for a larger, definitive clinical trial.

## Methods/Design

### Trial design

The Inpatient Suicide Intervention and Therapy Evaluation (INSITE) trial is designed as a single blind randomised controlled trial (RCT) with participants recruited from acute psychiatric wards from NHS trusts in the North West of England, UK. Sixty participants will be recruited and randomly allocated, using an independent source, to treatment as usual (TAU) or to TAU plus 20 sessions of Cognitive Behavioural Suicide Prevention Therapy (CBSP). TAU consists of medical and nursing care and medication as appropriate, plus multidisciplinary team management during inpatient stay. Following discharge, TAU would be variable depending on patients’ needs but typically would consist of multidisciplinary team management of community mental health team. Participants will be followed up at 6 weeks and 6 months (post intervention). The end of treatment is a fixed time and does not vary depending on treatment progress.

The project runs over three phases using a mixed-methods approach following the Medical Research Council’s (MRC’s) framework for developing complex interventions.

In phase 1, we conducted qualitative data collection from staff (*n* = 20) and patients (*n* = 20) investigating their views of inpatient care and on the implementation of CBSP, which has been used to inform the treatment and implementation protocol of the RCT. Phase 2 is the RCT evaluating CBSP reported here. Phase 3 will involve qualitative evaluation of staff and patient experiences and views of CBSP. A purposive sample of patients from both arms of the RCT will be sought to elicit a broad range of views regarding CBSP. This will include a balance of participants who have had a good outcome, those who have dropped out of the intervention and control participants. Interviews will explore participants’ and staff experiences and expectations of therapy, views on the content of CBSP and facilitators and barriers to implementation of the approach within inpatient settings. Informed consent will be obtained from all participants.

### Ethics and governance

The INSITE study is supported by the NIHR Research for Patient Benefit Programme (NIHR RfPB, grant number PB-PG-1111-26026). The study has been approved by the NRES Committee North West – Lancaster (registration number 13/NW/0504) and is conducted following guidelines of Good Clinical Practice (GCP) in accordance with the principles of the Declaration of Helsinki. The study is also being carried out in collaboration with the Samaritans and a local branch of Mind. Trial monitoring is carried out through the host trust, Manchester Mental Health and Social Care Trust, and the trial is overseen by an independent trial steering group consisting of academics, service users, carers and a statistician. In addition, a Service User Reference Group consisting of up to eight members who have previous experience of inpatient suicidality contribute to the design and execution of the trial. They provide monthly ongoing advice to the research team throughout the trial

### Inclusion criteria

Participants who are between the ages of 18 and 65 years of age, are current inpatients of an acute psychiatric ward, are able to provide informed consent, have experienced suicidal thoughts or behaviours within the 3 months prior to admission (as evidenced by ward staff report and confirmed by self-report and case note review following consent) and have sufficient English language capacity, are included.

### Recruitment and randomisation

Recruitment is over an 18-month period from May 2014 to the end of 2015. Eligible participants are identified by inpatient ward staff approached by trained research assistants or clinical studies officers from the Comprehensive Research Network (CRN). Participants deemed eligible to participate are provided with an information sheet detailing involvement in the trial and informed consent is taken 24 hours later should they wish to take part. Research assistants carry out a battery of assessments following which participants are randomly assigned to either TAU or TAU plus CBSP. Randomisation is statistician-led and pseudo-random, carried out using Sealed Envelope© Software [22] with group stratification by gender and history of self-harm. Allocation concealment is ensured as participant randomisation codes are not revealed until the participant is recruited into the trial. Participants are followed up by research assistants blinded to treatment allocation at 6 weeks and 6 months following baseline assessment. Procedures for maintaining blindness are developed for the trial, including steps and actions taken to ensure the blinding process is conducted correctly and to avoid jeopardising the blinding of the research assistant. During assessment participants are reminded by the research assistant to not disclose their treatment allocation. Blindness is monitored by the trial project coordinator who is not blinded to treatment and manages all follow-up appointments. Where blind breaches occur prior to follow-up appointments, subsequent data collection is allocated to an alternative research assistant to ensure all follow-up data is collected by blind assessors.

### Intervention

The intervention consists of TAU plus 20 CBSP sessions of up to 1-hour duration, over 6 months. Participants who are discharged during the treatment envelope continue their sessions in the community. TAU includes usual nursing and medical care during the inpatient stay, including medication and assessments, reviews and evaluation by the ward team. Following discharge from the ward, TAU is overseen by the appropriate care professional (e.g. care coordinator, GP, psychiatrist) and usually involves medical and multidisciplinary review and monitoring. Psychological therapies may also be offered to some participants as part of their TAU and this will be monitored.

CBSP will be delivered once or twice weekly during the participants’ inpatient stay and will continue at weekly intervals following discharge into the community. The intervention is guided by a detailed treatment protocol based on the original CBSP treatment protocol [19] but refined for use with inpatients by the project team and from data informed from pre-trial staff and patient qualitative interviews. CBSP is a recovery-based intervention, based on a detailed understanding of the individual’s experiences which aims to address and reduce the processes involved in ‘suicide schema’ activation, maintenance and elaboration using cognitive behavioural approaches. Therapy is carried out by clinical psychologists meeting the British Association of Behavioural and Cognitive Psychotherapies minimum standards for CBT practice. Treatment fidelity is maintained through adherence to a detailed treatment protocol and weekly supervision. In addition, therapy sessions are audio-recorded where permission to do so is granted. These are rated by the supervisory team, using the Cognitive Therapy Scale for Psychosis [23], to ensure therapists are adhering to CBT principles. Following the conclusion of the trial, a sample of recorded sessions will be subject to an independent fidelity check carried out by an external expert not involved in the trial.

### Primary outcome measures

The primary outcome is feasibility and acceptability of the intervention. Outcomes will be measured by uptake and attendance of therapy sessions during the inpatient stay and following discharge (a minimum of 10 sessions attended will be accepted as success), time spent in therapy, attrition (we anticipate a 20 % attrition rate based on similar RCTs with complex populations) and therapeutic alliance. The acceptability of the intervention and user views will be explored in phase 3 through semi-structured individual interviews with a purposive sample of participants and staff at the end of the intervention.

### Secondary outcomes

Secondary outcomes include measures of suicidal ideation, psychopathology, functioning, service use (to inform health economy data) and psychological variables associated with prediction of suicide:

1. Measures of the intensity and duration of suicidal ideation, and suicidal plans and behaviour have been selected to reflect the continuum of suicidality from thoughts through to behaviours. Each of the measures will be completed at baseline, 6 weeks and 6 months following baseline assessment (end of treatment):
  - *The Beck Scale for Suicidal Ideation (BSSI)* is a 21-item self-report scale evaluating suicidal ideation, planning and intent over the last week [24]. The scale has good reliability and validity [24]
  - *The Suicidal Behaviours Questionnaire-revised (SBQ-R)* is a 4-item measure used extensively in clinical settings to establish risk of suicide, revised to report on suicidality in the past 3 months [25]. The scale has good reliability and validity in clinical and non-clinical samples [25]
  - *The Suicide Probability Scale (SPS)* is an 18-item self-report scale indicating further suicide behaviour probability. The scale has good internal consistency and test-retest reliability [26]
  - *The Beck Hopelessness Scale (BHS)* is a 20-item self-report tool, measuring negative beliefs about the future over three domains of hopelessness over a week [27]. Total scores range from 0–20 with higher scores indicating higher levels of hopelessness and is widely used in clinical settings. There is strong evidence for the scale’s convergent validity and internal consistency in clinical populations [28]
  - *Review of Clinical Records.* This review will be conducted by a member of the research team to identify number of episodes of suicidal behaviour from the case records
2. Psychopathology:
  - *The Positive and Negative Syndrome Scale (PANSS)* [29], is an interviewer-based scale which includes scales of positive symptoms, negative symptoms and general psychopathology and is used widely in mental health research. The scale has good internal reliability and concurrent validity [30]
  - *The Psychotic Symptoms Rating Scales (PSYRATS)* is a well-validated assessment of the frequency and intensity of hallucinations and delusions in psychosis and associated distress. The scale has excellent psychometric properties with inter-rater reliability for the scales ranging between 0.78–1.0 [31]
  - *The Calgary Depression Scale (CDS)* [32] is a 9-item observer-rated measure specifically designed for people with severe mental health problems, minimising contamination by negative symptoms and the extrapyramidal side effects of neuroleptics. The scale has high inter-rater reliability and discriminant validity [33]
  - *Sleep Condition Indicator (SCI)* is an 8-item scale measuring quality of sleep over the period of a month. Sleep disturbances have been linked to a number of psychopathologies. The SCI is valid, reliable and sensitive to change in insomnia severity [34]
3. Functioning:
  - *Personal and Social Performance Scale (PSP)* is an interviewer-rated scale which assesses personal and social functioning over four domains via a semi-structured interview [35]. The scale has adequate internal consistency reliability and is sensitive to differences in social functioning [36]
4. Quality of life:
  - *WHO Quality of Life-Brief (WHOQOL-Brief)* is 26-item self-report scale which measure the following domains: physical health, psychological health, social relationships and environment [37]. Confirmatory factor analyses of internal consistency, item-total correlations, discriminant validity and construct validity indicate that the scale has good to excellent psychometric properties of reliability [38]
5. Negative self-appraisals and other psychological variables associated with suicide:
  - *The Defeat Scale* is a 16-item scale assessing defeat, failed struggle and low social rank over the last 7 days [39]
  - *The Entrapment Scale* is a 16-item, self-report scale assessing feelings of being trapped by internal and external events in the past 7 days. The entrapment and defeat measures have good psychometric properties and significantly correlate with depression [39]
  - *The Self-Concept Questionnaire (SCQ)* is a self-report measure of seven components of self-esteem using 30 items [40]. Investigation of convergent and discriminant validity are encouraging [40]. The SCQ has high reliability and good concurrent and discriminant validity [41]
  - *Coping in Stressful Situations (CSS)* is a 48-item measure that assesses three types of coping styles [42]. The inventory has excellent psychometric properties [42, 43]
6. Service user and staff perceptions of acute inpatient wards (at baseline and 6 months only):
  - *Views on Inpatient Care (VOICE-Patient measure)* is a 19-item questionnaire exploring service users’ experience of inpatient wards. The measure has good validity and internal and test-retest reliability [44]
  - *Views on Therapeutic Environments (VOTE-Staff measure)* will be completed by staff pre and post intervention and is implemented on the wards. A 20-item questionnaire exploring the perceptions of staff on inpatient wards, the scale has good test-retest concordance, strong internal consistency, criterion, face and content validity [45]
7. Data required for cost analysis on service use:

- *EuroQol five dimensions (EQ-5D)* is a NICE-recommended measure that documents health status required for quality-adjusted life-year (QALY) estimation for primary economic analysis. It assesses five domains of mobility, self-care, usual activity, pain/distress, and anxiety/depression [46]. Health status profiles will be converted into utility values using utility tariffs for EuroQol to estimate QALYs [47]
- *Use of Services Inventory* is a trial specific data capturing form to collect data on service usage during the study
- *Case notes review* will be conducted to collect data on service use for each participant to estimate costs. Based on previous work in mental health, data on service use for 3 months prior to entry to the study will be collected

### Therapeutic alliance and treatment fidelity

The therapeutic alliance and engagement in therapy will be measured using the *Working Alliance Inventory (WAI) (client and therapist version)* [48]*,* administered at session 4 and post therapy. The WAI is a self-report measure of the therapeutic bond, task agreement and goal agreement with higher scores indicating a better therapeutic alliance between client and therapist.

### Data analysis

In accordance with the Consolidated Standards of Reporting Trials (CONSORT) principles [49], we will report all participant flow. Demographic data will be described using summary statistics (means and standard deviations or number and percentage).

The main efficacy analysis is on an intention-to-treat basis with data from all participants. Every effort will be made to follow up all participants in both arms for assessments and the analysis will use, where appropriate, statistical techniques for handling missing data. Descriptive statistics will be used to summarise the primary outcome measures.

The secondary outcome measures will be analysed using a linear regression model, allowing for the baseline measurement of outcome, treatment assignment and NHS trust as covariates, at each assessment point separately. The coefficient of the treatment assignment is an estimate of the between-group treatment effect, and can inform potential effect sizes for a future definitive trial. Point estimates and associated 95 % confidence intervals will be reported rather than tests of statistical significance (*p* values).

### Economic evaluation

A cost-effectiveness analysis will be performed to further investigate the impact of CBSP for patients and health and social care providers. Data on costs, service use and health status will be collected. Unit costs for each type of service will be derived from local and national databases and statistics. The main outcome of interest for a cost-effectiveness analysis is the likely cost-effectiveness of the CBSP intervention. Secondary analysis of the data will explore: the structural uncertainty associated with the trial design; the extent to which EQ-5D scores and QALY values are correlated with clinical outcome measures and discriminate between groups and sources of external data. Baseline covariates in patient clinical and socioeconomic characteristics and pre-study service use will be controlled for. Factors known to influence costs and QALYs (e.g. ethnicity, socioeconomic status) will be collected at baseline to statistically control for their impact. The data will also be used to inform sample size decisions for a definitive trial.

### Qualitative data

All interviews will be digitally audio-taped and transcribed verbatim. An inductive approach using Thematic Analysis [50] will be taken to elicit patterns within the data corpus using standard procedures involving identifying meaning codes and categories at both the semantic and latent level [50]. Data generation and analysis will continue in parallel, using the constant comparative technique. Disconfirming evidence will be sought, until thematic category saturation is achieved. Records of field notes will be maintained and reflections providing adjunctive data will be used to illuminate and justify interpretative decisions.

Analysis will be primarily conducted by the experienced qualitative applicants, but interim analysis will involve the wider multi-disciplinary research team, including the study’s service user reference group (SURG). Integrating data from different stakeholders, and researcher triangulation increases the trustworthiness of the final analysis [48].

## Discussion

The strength of the INSITE pilot RCT is that it will be one of the first to investigate the use of psychological therapies in acute psychiatric wards including a range of outcomes. It will explore key issues relating to experiences of suicidal patients and pragmatic issues that will inform a larger, definitive RCT that will examine the effectiveness of CBSP in inpatient settings, treatment uptake and recruitment. A limitation of the study design includes insufficient power to determine the effectiveness of key suicidality outcomes. The study sample was recruited from one mental health trust which is not representative of the wider inpatient environment.

Some of the issues that will need to be addressed include whether it is possible to recruit sufficient numbers of participants for a full-scale trial and to identify possible reasons why patients may, or may not, want to participate in the trial. We will discover if it is possible to retain participants in the intervention, both during their inpatient care and following discharge into the community. NHS services are often criticised for failings in the continuity of care, particularly for discharge from acute psychiatric wards when there is an increased risk of suicide [1, 15]. Therapy that begins on the ward and continues into the community may help to avoid such discontinuity. Working on the ward presents several challenges in itself and there are few precedents of psychological therapy being delivered in acute psychiatric settings. The study will investigate what features of ward activity need to be considered to successfully implement this intervention. CBSP has been employed in a community setting and for prisoners, but this study will examine how the therapy should be modified for acute inpatients.

It is also necessary to consider the most effective and sensitive outcome measures to detect meaningful change resulting from CBSP. Existing instruments measuring suicidal behaviours and ideas have limitations and may require refinement, particularly to reflect service-user defined outcomes of recovery from suicidality. The study would like to explore what training and support is required to maximise the contribution of service users to suicidality research. In order to determine whether CBSP is value for money, we will require data depicting the cost of current treatment to compare it to. Evidence of value for money is required to inform future NHS policy and funding decisions regarding CBSP. This information will have to be comprehensive to affect resource allocation. In addition to the research aims outlined, we are interested in the impact that the introduction of CBSP had on a number of patient and staff outcomes, including: patient experience of therapy; impact on suicidality; and ward staff views on the use of CBSP in the inpatient setting.

## Trial status

We are currently recruiting participants for our trial.

## Additional file

Additional file 1:
**CONSORT 2010 checklist of information to include when reporting a randomised trial*.** (DOC 218 kb)

## Abbreviations

BHS: Beck Hopelessness Scale
BSSI: Beck Scale for Suicidal Ideation
CBSP: Cognitive Behavioural Suicide Prevention
CBT: Cognitive Behavioural Therapy
CDS: Calgary Depression Scale
CSS: Coping in Stressful Situations
EQ-5D: EuroQol
GCP: Good Clinical Practice
GP: General Practitioner
CRN: Comprehensive Research Network
INSITE: Inpatient Suicide Intervention and Therapy Evaluation
NICE: National Institute for Health and Care Excellence
NIHR: National Institute for Health Research
PANSS: Positive and Negative Syndrome Scale
PSP: Personal and Social Performance Scale
PSYRATS: Psychotic Symptoms Rating Scales
QALY: Quality-Adjusted Life Year
RCT: Randomised Controlled Trial
SAMS: Schematic Appraisal Model of Suicide
SBQ-R: Suicidal Behaviours Questionnaire-revised
SCI: Sleep Condition Indicator
SCQ: Self-Concept Questionnaire
SPS: Suicide Probability Scale
TAU: Treatment As Usual
VOICE: Views on Inpatient Care
VOTE: Views on Therapeutic Environment
WAI: Working Alliance Inventory
WHOQOL-Brief: World Health Organisation Quality of Life-Brief
